# Broad-spectrum humanized monoclonal neutralizing antibody against SARS-CoV-2 variants, including the Omicron variant

**DOI:** 10.3389/fcimb.2023.1213806

**Published:** 2023-08-14

**Authors:** Kun Wen, Jian-Piao Cai, Xiaodi Fan, Xiaojuan Zhang, Cuiting Luo, Kai-Ming Tang, Huiping Shuai, Lin-Lei Chen, Ricky Ruiqi Zhang, Jianwen Situ, Hoi-Wah Tsoi, Kun Wang, Jasper Fuk-Woo Chan, Shuofeng Yuan, Kwok-Yung Yuen, Hongwei Zhou, Kelvin Kai-Wang To

**Affiliations:** ^1^ Microbiome Medicine Center, Division of Laboratory Medicine, Zhujiang Hospital, Southern Medical University, Guangzhou, China; ^2^ State Key Laboratory for Emerging Infectious Diseases, Carol Yu Center for Infection, Department of Microbiology, School of Clinical Medicine, Li Ka Shing Faculty of Medicine, Pokfulam, The University of Hong Kong, Hong Kong, Hong Kong SAR, China; ^3^ Guangdong Provincial Key Laboratory of Tumor Interventional Diagnosis and Treatment, Zhuhai People’s Hospital (Zhuhai Hospital Affiliated with Jinan University), Zhuhai, China; ^4^ Department of Microbiology, Queen Mary Hospital, Pokfulam, Hong Kong SAR, China; ^5^ Department of Clinical Microbiology and Infection Control, The University of Hong Kong-Shenzhen Hospital, Shenzhen, China; ^6^ Center for Virology, Vaccinology and Therapeutics, Hong Kong Science and Technology Park, Hong Kong, Hong Kong SAR, China

**Keywords:** SARS-CoV-2, monoclonal antibody, therapeutics, prophylaxis, variants of concern

## Abstract

**Introduction:**

Therapeutic monoclonal antibodies (mAbs) against the SARS-CoV-2 spike protein have been shown to improve the outcome of severe COVID-19 patients in clinical trials. However, novel variants with spike protein mutations can render many currently available mAbs ineffective.

**Methods:**

We produced mAbs by using hybridoma cells that generated from mice immunized with spike protein trimer and receptor binding domain (RBD). The panel of mAbs were screened for binding and neutralizing activity against different SARS-CoV-2 variants. The *in vivo* effectiveness of WKS13 was evaluated in a hamster model.

**Results:**

Out of 960 clones, we identified 18 mAbs that could bind spike protein. Ten of the mAbs could attach to RBD, among which five had neutralizing activity against the ancestral strain and could block the binding between the spike protein and human ACE2. One of these mAbs, WKS13, had broad neutralizing activity against all Variants of Concern (VOCs), including the Omicron variant. Both murine or humanized versions of WKS13 could reduce the lung viral load in hamsters infected with the Delta variant.

**Conclusions:**

Our data showed that broad-spectrum high potency mAbs can be produced from immunized mice, which can be used in humans after humanization of the Fc region. Our method represents a versatile and rapid strategy for generating therapeutic mAbs for upcoming novel variants.

## 1 Introduction

Severe acute respiratory syndrome coronavirus 2 (SARS-CoV-2), especially the Omicron variant, causes mild disease in most patients (Chan et al., 2020a; To et al., 2021). However, in older adults or patients with comorbidities, SARS-CoV-2 infection can cause severe disease with high case-fatality rate (Chen et al., 2022a). Several treatment strategies have been associated with improved clinical outcome. Drugs that modulate the inflammatory response, including dexamethasone, interferon-β-1b, and baricitinib, have been shown to improve clinical outcomes with a significantly shorter time to symptom resolution in randomized clinical trials (Hung et al., 2020; Group et al., 2021; Kalil et al., 2021). Remdesivir, a viral RNA polymerase inhibitor, is the first antiviral that was shown to have beneficial effect (Goldman et al., 2020). Molnupiravir and nirmatrelvir-ritonavir are oral antivirals which were shown to reduce progression to severe disease among non-hospitalized patients (Hammond et al., 2022; Jayk Bernal et al., 2022).

Monoclonal antibodies (mAbs) targeting the SARS-CoV-2 spike protein have also been used for treatment (Corti et al., 2021). These mAbs have been divided into seven categories based on the binding sites (Hastie et al., 2021). Mutations in the spike (S) protein, especially in the receptor binding domain (RBD), render many mAbs ineffective against novel variants. For example, the Beta and Gamma variants are resistant to Casirivimab, Bamlanivimab and Etesevimab (Hwang et al., 2022), while the Omicron variant BA.1 sublineage is resistant to most commercially available mAbs except Bebtelovimab (Takashita et al., 2022).

In this study, we identified a broad-spectrum mAb, WKS13, which can potently neutralize different SARS-CoV-2 variants. Both murine and humanized versions of WKS13 could protect hamsters from viral challenge with Delta variant.

## 2 Methods

### 2.1 Cell lines

NS1 (ATCC TIB-18TM) was used for hybridoma fusion. ExpiSf9 [Thermo Fisher Scientific Inc.; Cat# A35243], Sf9 [Thermo Fisher Scientific Inc.; Cat# 11496015] and Expi293F™ [Thermo Fisher Scientific Inc.; Cat# A14528]) were used in the generation of recombinant proteins. BHK21 (ATCC CCL-10™) was used for the generation of pseudovirus. HEK293T-ACE2 [GeneCopeia Cat# SL221]) was used for pseudovirus neutralization test (pVNT). VeroE6/TMPRSS2 cells (JCRB cell bank of Okayama University; Cat# JCRB1819) was used for conventional live virus neutralization test (cVNT). All cell lines used in this study were routinely tested for mycoplasma and found to be mycoplasma-free.

### 2.2 Preparation and identification of mAbs

Six-week-old female BALB/c mice (n=2) were immunized intramuscularly with S protein trimer of ancestral SARS-CoV-2 at 10 μg/mice (Acro Biosystems, Newark, USA; Cat# SPN-C52H9), which was mixed with an equal volume of QuickAntibody adjuvant (Biodragon Immunotechnologies Co., Ltd. Beijing, China; Cat# KX0210042), on day 0 and day 21, and then boosted intraperitoneally with 100 μg of recombinant RBD (Acro Biosystems, Newark, USA; Cat# SPD-C52H3) of ancestral SARS-CoV-2 at Day 35. On day 38, spleen was harvested. Hybridoma cells were produced by fusing splenic cells from immunized mice with myeloma cells and were selected using hypoxanthine-aminopterin-thymidine (HAT) medium. Hybridoma clones were screened for the presence of mAbs against SARS-CoV-2 by indirect enzyme immunoassay (EIA) with recombinant ancestral S protein as coated antigens. Cell subclones secreting the specific mouse mAbs were further selected by limiting dilution of positive hybridoma cells. Hybridoma cells specific for SARS-CoV-2 S protein were then injected intraperitoneally in mice, and the ascitic fluid were collected 10 days after inoculation. The mAb in the ascitic fluid was purified by precipitating with caprylic acid and ammonium sulfate. The mAb isotype was determined with the Mouse Monoclonal Antibody Isotyping Kit (Biodragon Immunotechnologies Co. Beijing, China; Cat# BF16002). The antibody titer was determined as the lowest antibody concentration that produced a positive test reaction by EIA against recombinant RBD protein.

### 2.3 Antibody binding kinetics and epitope binding (competition) assays

The binding affinity of mAbs was determined using the biolayer interferometry (BLI) (Gator™ System, Gator Bio, Suzhou, China). Anti-mouse IgG Fc biosensors were pre-equilibrated in Q buffer and captured 10 μg/ml of mAbs for 240 s. After equilibrating in Q buffer for another 60 s, the sensors were incubated with serially-diluted recombinant ancestral RBD protein or Q buffer (as control) for 180 s, and finally dissociated in Q buffer for 300 s. Gator™ System Analysis software was used to calculate association (Ka) and dissociation (Kd) kinetic rate constants, and equilibrium dissociation constant (KD) of these mAbs.

We next determined the epitopes of these anti-RBD antibodies using a competition assay by BLI as described (Dong et al., 2020). Anti-His biosensors were pre-equilibrated in Q buffer and captured 10 μg/ml of recombinant His-tagged RBD protein for 180 s, followed by binding with 10 μg/mL of the primary antibody for 300 s and then the secondary antibody (or buffer control) for another 300 s. The wavelength shift of the primary and the secondary antibody on the probe surface were recorded and the competition rates between these two antibodies were calculated as following: % Competition = (1- (Shift_1st_ – Shift_2nd_)/(Shift_1st_ - Shift_Control_)) × 100. The graph was generated using the GraphPad Prism software.

### 2.4 RBD expression of ancestral strain and variants

Recombinant RBDs (residues 306-543) of ancestral strain and variant SARS-CoV-2 spike proteins were expressed and purified in insect cells as we described previously (Chen et al., 2022b). The ancestral RBD were designed based on the reference sequence Wuhan-Hu-1 (GenBank ID YP_009724390.1). The RBD mutants are listed in **Supplementary Table S1**. Briefly, RBD gene sequences were baculovirus-codon-optimized and cloned into pFast dual baculovirus expression vector (Thermo Fisher Scientific, Waltham, MA, USA Cat # 10712024). The constructs were fused with an N-terminal gp67 signal peptide and C-terminal 6× His tag for secretion and purification. A recombinant bacmid DNA was generated using the Bac-to-Bac system (Thermo Fisher Scientific, Waltham, MA, USA). Baculovirus was produced by transfecting purified bacmid DNA into Sf9 cells using Cellfectin (Thermo Fisher Scientific, Waltham, MA, USA; Cat# 10362100), and subsequently used to infect ExpiSf9 cell suspension culture at a multiplicity of infection of 1 to 10. Infected ExpiSf9 cells were incubated at 27.5 °C with shaking at 125 r.p.m. for 96 hours for protein expression. The supernatant was collected and then concentrated using a 10 kDa MW cutoff Lab-scale TFF System (Millipore, Burlington, USA). The RBD protein was purified by Ni-NTA purification system, followed by size exclusion chromatography, and buffer exchanged into 1× phosphate buffered saline (PBS) pH 7.4. The concentration of purified RBD was determined by using the Bradford Assay Kit (Bio-Rad, California, USA; Cat# 5000002) according to the manufacturer’s instructions.

### 2.5 Enzyme immunoassay measuring antibody against S protein and RBD

EIA for anti-S and anti-RBD mAbs was performed as we described previously with modifications (To et al., 2021). Briefly, 96-well Immuno plates (Nunc, Roskilde, Denmark; Cat# 243656) were coated with 100 μL/well (0.1 μg/well) of His-tagged SARS-CoV-2 S or RBD in 0.05 M carbonate bicarbonate buffer (pH 9.6) overnight at 4°C and then followed by incubation with a blocking buffer. After blocking, mAbs were diluted in a series of 2-fold dilution from 1 μg/ml. Then 100 μl diluted mAbs was added to the wells and incubated at 37°C for 1 hour. The attached mouse IgG was detected using horseradish-peroxidase-conjugated goat anti-mouse IgG antibody (Invitrogen, Thermo Fisher Scientific, Waltham, MA, USA; Cat# 31430). The reaction was developed by adding diluted 3,3’,5,5’-tetramethylbenzidine single solution and stopped with 0.3 N H_2_SO_4_. The optical density (OD) was read at 450 and 620 nm.

### 2.6 S-RBD/hACE2 blocking assay

The neutralizing activity of the ten selected mouse mAbs was evaluated by detecting their inhibition for the binding of recombinant ancestral RBD protein to its human angiotensin-converting enzyme 2 (hACE2) receptor (Acro Biosystems, Newark, USA; Cat # AC2-H52H8) by BLI with Gator™ Label-Free Analysis System. All experiments were carried out at 25°C. Anti-His biosensors were first pre-equilibrated in Q buffer containing PBS (10 mM pH 7.4), 0.02% Tween-20, and 0.2% bovine serum albumin (BSA) for 300 s, and 1 μg/ml recombinant ancestral RBD of SARS-CoV-2 S protein was loaded onto these probes for 200 s. After equilibrating in Q buffer for another 30 s, the probes were incubated with serially diluted anti-RBD mAbs or dilution buffer (as control) for 180 s and followed by binding to 2 μg/ml of recombinant hACE2 protein for 180 s. The wavelength shift (indicative of binding signal) of hACE2 protein on the probe surface was recorded and the inhibition efficiency of the anti-RBD mAbs were calculated as follows: % Inhibition= (1- Shift_mAbs_/Shift_Control_) × 100. The IC_50_ was calculated using log(inhibitor) vs. response (3 parameters).

### 2.7 Virus culture and conventional live virus neutralization test

Viral culture and conventional live virus neutralization test (cVNT) were performed in the Biosafety Level 3 facility at the University of Hong Kong as we described previously (Chu et al., 2020; Lu et al., 2022). In order to avoid mutations, viral culture was conducted using VeroE6/TMPRSS2 cells. Briefly, mouse mAbs were serially diluted in 2-folds starting from 200 µg/ml, while humanized WKS13 mAb was serially-diluted in 2-folds starting from 30 μg/ml. Then, 100 TCID_50_ of virus isolates were mixed with mAb and incubated for 1 hour. Finally, 100 μl mAb-virus mixture was then added to VeroE6/TMPRSS2 cells. After incubation for 3 days, cytopathic effect was visually scored. All dilutions were performed in duplicates. The neutralization EC_50_ titers were plotted using 5-parameter nonlinear fit in GraphPad Prism version 9.1.1. For mouse mAbs, a value of 0.12 μg/ml was assigned if no cytopathic effect was seen at a concentration of 0.24 μg/ml. For humanized WKS13, a value of 30 μg/ml was assigned if cytopathic effect was seen at a concentration of 15 μg/ml.

### 2.8 Production and titration of pseudoviruses

The spike gene of the Wuhan-Hu-1, Alpha, Beta, Delta, Gamma, and Omicron variant BA.1, BA.2, BA.2.75.2 and BA.5 sublineages were used for pseudovirus generation. The spike mutants are listed in **Supplementary Table S2**. The spike gene was codon-optimized and cloned into the eukaryotic expression plasmid pCAGEN (Addgene, Watertown, Massachusetts, USA; Cat# 11160) to generate a vesicular stomatitis virus (VSV) pseudovirus plasmid pCAG-SARS-CoV-2-S as previously described (Chan et al., 2022a). BHK21 cell was transfected with spike plasmid using Lipofectamine 3000 (Thermo Fisher Scientific, Waltham, MA, USA; Cat# L3000015) following manufacturer’s instruction. After 24 hours, transfected cells were infected with VSV-ΔG/G*-luciferase. One hour after incubation, infected cells were washed three times with PBS, anti-VSV-G antibody (1 μg/ml) was added to neutralize excess VSV-ΔG/G*-luciferase. Twenty-four hours post-infection, culture supernatant was harvested and stored at −80°C in 0.5 ml aliquots until use. Serial 2-fold dilution of each pseudovirus was inoculated onto HEK293T-ACE2 cells on 96-well plates. After 24 h incubation, the culture supernatant was removed, and cell lysis buffer was added to lyse the cells for 15 min. Cell lysate was mixed with firefly luciferase substrate (Promega, Madison, USA; Cat# E4550) and transferred to White Opaque 96-well Microplate (PerkinElmer, Waltham, USA; Cat# 6005299) for the detection of luminescence by GloMax Explorer (Promega, Madison, USA).

### 2.9 Pseudovirus neutralization assay

HEK293T-ACE2 cells were seeded in a 96-well plate at a density of 5 × 10^4^ cells/well 24 h before assay. Each mAb was 4-fold diluted for 6 times, starting from 100 μg/ml. Each diluted mAb was mixed with 1 × 10^6^ RLU/ml rVSV-ΔG/S*-luciferase pseudoviruses. A mAb against *Talaromyces marneffei* Mp1p protein was used as a negative control (Wang et al., 2011). The mixture was incubated at 37°C for 1 h before being added to cells in 96-well plates. Twenty-four hours after infection, all samples were lysed and analyzed for luciferase readouts according to the manufacturer protocol. The inhibition of luciferase activity from each serum dilution could be calculated as follows: 100 – [(mean RLU from each sample (virus + serum) - mean RLU from CC)/(mean RLU from VC-mean RLU from CC) × 100]. The IC_50_ (the half-maximal inhibitory concentration) values was calculated using nonlinear regression (log [inhibitor] vs response [four parameters]), with GraphPad Prism Version 9.1.1.

### 2.10 SARS-CoV-2 challenge in Golden Syrian hamster model

Female Syrian hamsters (4–6 weeks old) were obtained from the Chinese University of Hong Kong Laboratory Animal Service Centre through the University of Hong Kong (HKU) Centre for Comparative Medicine Research. The experimental procedures were approved by the Animal Ethics Committee on the Use of Live Animals in Teaching and Research of HKU (CULATR 5370-20) and were performed according to the standard operating procedures of Biosafety Level 3 animal facilities (Chan et al., 2020b; Chan et al., 2020c). Briefly, hamsters were intranasally inoculated with 10^5^ plaque-forming units (p.f.u.) SARS-CoV-2 Delta strain (B.1.617.2) (hCoV-19/Hong Kong/HKU-210804-001/2021; GISAID: EPI_ISL_3221329) as previously described (Chan et al., 2020b; Yuan et al., 2022). 24 hours after infection, the hamsters were either administered intraperitoneally with 5 mg/kg mouse WKS13, 5 mg/kg humanized WKS13, or equal volume of PBS. Three days later, one mouse WKS13-treated group, one humanized WKS13-treated group, and one control group were sacrificed for virological and histopathological analyses as previously described (Chan et al., 2022b), the other two groups were kept for body weight monitoring. The right lung was used for the determination of viral titer by using plaque assay, while the left lung was used for histopathological analysis and immunofluorescence staining (Lee et al., 2020).

### 2.11 Determination of viral load using plaque reduction assay

Plaque reduction assay was performed in a 24-well tissue culture plates as we described previously with slight modifications (Yuan et al., 2021). Briefly, hamster lung was lysed by magnetic beads in 1 ml Dulbecco’s Modified Eagle Medium (DMEM), and then diluted 1:100, 1:1000 and 1:10000 with DMEM. The lung homogenate at different dilutions was added to VeroE6/TMPRSS2 cells that were seeded in 24-well plates 24h before infection. The plates were further incubated for 2 h at 37°C in 5% CO_2_ before removal of unbound viral particles and washing once with DMEM. The cell monolayers were then overlaid with media containing 1% low melting agarose (Cambrex, East Rutherford, NJ, USA) in DMEM and incubated as above for 72 h. Next, the wells were fixed with 10% formaldehyde overnight. After removal of the agarose plugs, the cell monolayers were stained with 0.7% crystal violet, and the plaques were counted.

### 2.12 Histology and immunofluorescence staining

Fixed hamster lung tissues were processed, embedded, and cut to prepare 5 mm thick tissue sections on glass slides. Before staining, the slides went through dewaxed with xylene and serially decreased ethanol concentrations (100%, 95%, 70%). The tissue slides were stained with Gill’s hematoxyline and eosin (H&E) (Thermo Fisher Scientific, Waltham, MA, USA; Cat# 34002) as we previously described (Zhang et al., 2021).

For immunofluorescence staining, antigen retrieval of tissue slides were performed using Antigen Unmasking Solutions (Vector Labs, Newark, USA; Cat# H-3300-250). The slides were then incubated with an in-house rabbit antiserum against the SARS-CoV-2 nucleocapsid (N) protein. After incubation for 1 hour at room temperature, unbound antibodies were removed by washing with PBS-T six times. The bound anti-N antibodies were then detected by Alexa Fluor 488-conjugated goat anti rabbit IgG (H+L) cross-absorbed secondary antibody (Thermo Fisher Scientific, Waltham, MA, USA; Cat# A-11008) for 30 min at room temperature. After washing six times with PBST, the stained cells were mounted onto glass slides with VECTASHIELD mounting medium with 40,60-diamidino-2-phenylindole (DAPI) (Vector Labs, Newark, USA; Cat# H-1500-10). Images were acquired with the Olympus BX53 light microscope using a 20× objective.

### 2.13 Cloning and expression of recombinant humanized WKS13 monoclonal antibody

Total RNA was extracted from Trizol-lysed WKS13 hybridoma cell lines and amplified by RT-PCR with primers in the constant antibody regions. The PCR products were sequenced by Genewiz (Azenta life sciences, Chelmsford, USA). The sequences of WKS13 antibody heavy and light chain V genes (VH/VL) were synthesized (Sangon Biotech, China, Shanghai) and cloned into the human IgG1 expression vector with restriction endonucleases *AfeI* and *NheI* (New England Biolabs, Ipswich, MA. USA; Cat# R0652L and R3131L) for the heavy chain, and *AfeI* and *BsiWI* (New England Biolabs, Ipswich, USA; Cat# R0553L) for the light chain. Equal amounts of heavy chain and light chain plasmids were transfected into Expi293F cells using 1 mg/ml polyethylenimine (PEI) (Polysciences, Inc. Warrington, USA; Cat# 9002-98-6, 26913-06-4), with cells grown to a density of 3 × 10^6^ cells/mL. Following transfection, cells were maintained in Expi293™ Expression Medium (Thermo Fisher Scientific, Waltham, MA, USA; Cat# A1435103) with GlutaMAX™ Supplement (Thermo Fisher Scientific, Waltham, MA, USA; Cat# 35050061) at 37°C in a humidified 8% CO_2_ incubator rotated at 120 rpm. Six days after transfection, cell supernatant was harvested and clarified by centrifugation, and filtered through 0.22 µm filters and purified using chromatography cartridge protein A/G at room temperature. After washing with PBS, the antibodies were eluted from the chromatography cartridge protein A/G in chromatography columns using 0.1 M Na-Citrate (pH 3.0) and neutralized with 1M Tris-HCl (pH 8.8), followed by size exclusion chromatography and buffer exchanged into 1× PBS pH 7.4. The concentration of purified antibodies was determined by using the Bradford Assay Kit. The purity of recombinant mAb was verified using sodium dodecyl sulfate polyacrylamide gel electrophoresis (SDS-PAGE).

### 2.14 Statistical analysis

Statistical analysis was performed using PRISM version 9.1.1. Statistical comparisons between two experimental groups were performed using unpaired two-tailed Student’s t-tests. Comparisons among three or more experimental groups were performed using one-way or two-way ANOVA with Tukey’s multiple-comparison test. AUC values were calculated and analyzed using one-way ANOVA. Differences were considered to be statistically significant when P < 0.05.

## 3 Results

### 3.1 Generation and identification of anti-S monoclonal antibodies

**Figure 1:**
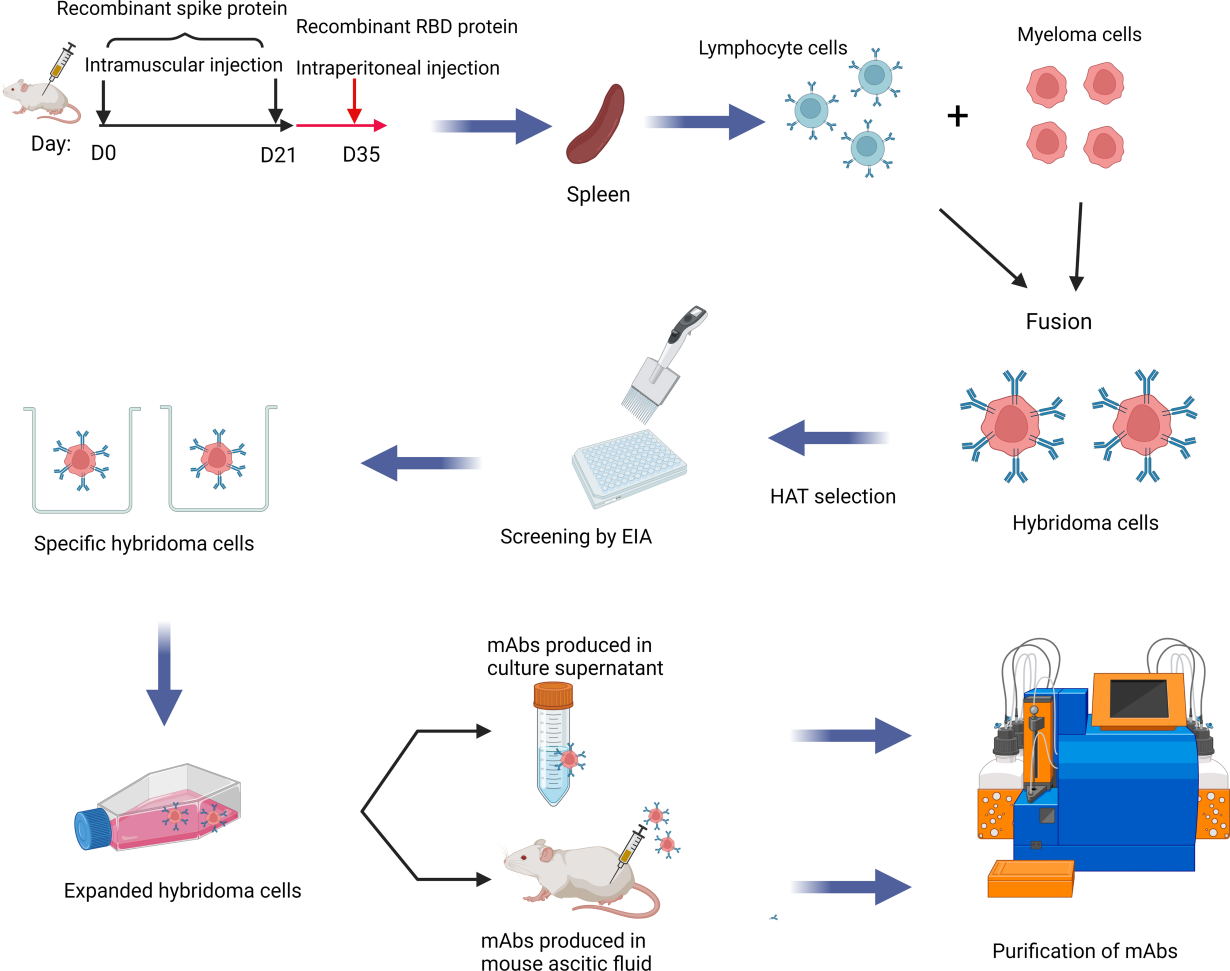
Schematic diagram showing the production of mAb in this study. mAbs were generated by immunizing BALB/c mice with spike and RBD protein of SARS-CoV-2 sequentially. Splenic cells and myeloma cells were fused and then selected in the HAT medium. Finally, hybridoma cells producing antibodies against the spike protein or the RBD were selected. This Graphical abstract was created with BioRender software (https://biorender.com). HAT, hypoxanthine-aminopterin-thymidine; EIA, enzyme immunoassay; mAbs, monoclonal antibodies.

To generate mAbs against the S protein, we first immunized two mice with ancestral S protein trimer on day 0 and day 21, and boosted with ancestral RBD protein on day 35. Splenic cells were isolated to generate hybridomas and produce mAbs (**Figure 1**). Cell subclones that produced antibodies against the S protein were selected using an EIA assay. We have screened a total of 960 clones. Eighteen mAbs with high OD values in EIA were selected, including 10 IgG1, 4 IgG2a, 3 IgG2b, and 1 IgG3. (**Supplementary Table S3**). Ten out of these 18 mAbs could bind to RBD.

### 3.2 Neutralizing activity and RBD-hACE2 blocking activity of mAbs

**Figure 2:**
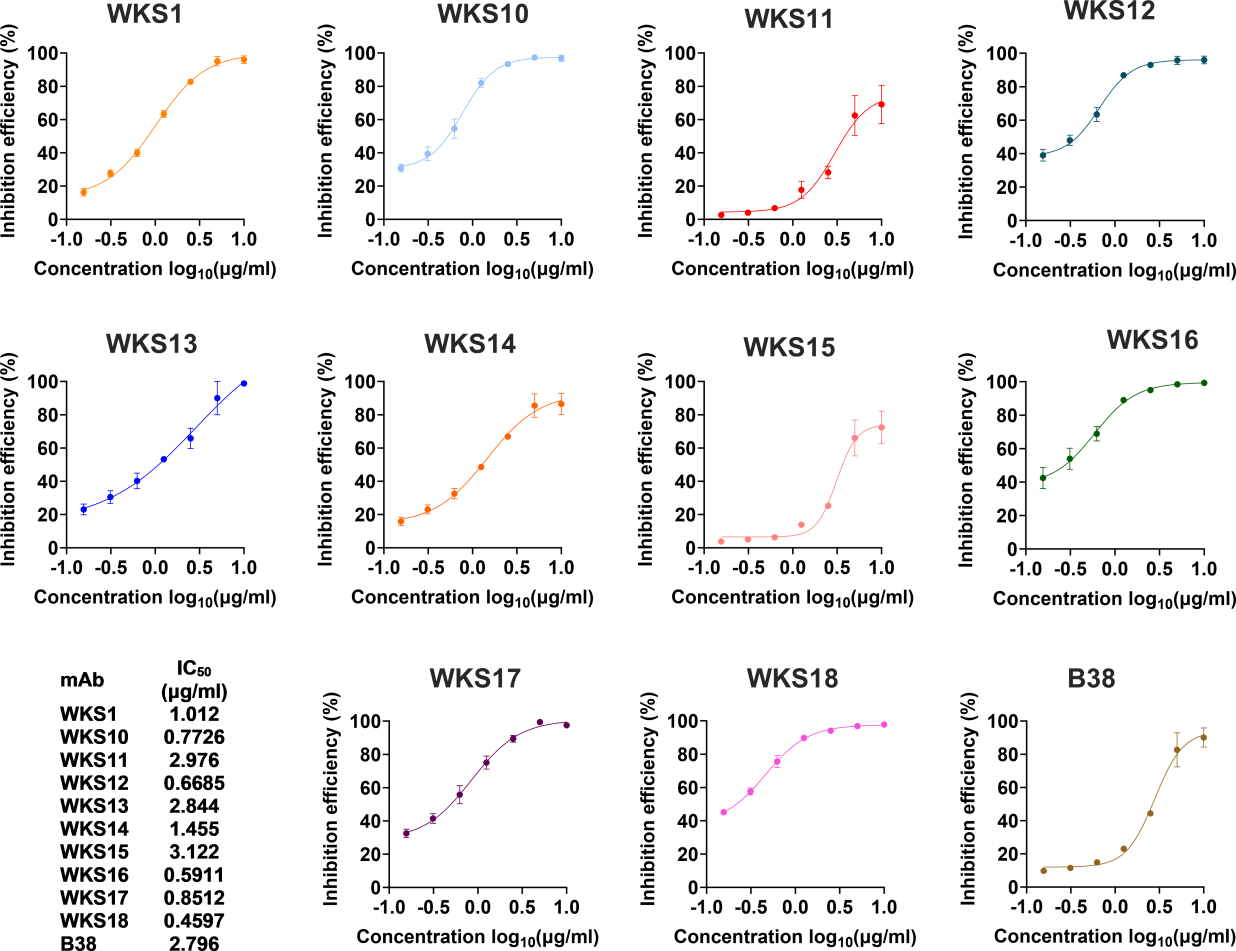
The inhibition of binding between the recombinant ancestral RBD protein and the human ACE2 receptor by different mAbs. The binding between the recombinant ancestral RBD protein and the human ACE2 receptor was measured using the biolayer interferometry. The previously published mAb (B38) was used as a control.

Next, we determined the neutralizing activity of the 10 RBD-binding mAbs and the 8 remaining spike mAbs that bind outside RBD. In the cVNT, 5 of 18 mAbs (WKS10, WKS12, WKS13, WKS16 and WKS18) were able to neutralize ancestral virus at a concentration of 3.125 μg/ml (**Supplementary Table S3**). The RBD-hACE blocking assay showed that all 10 RBD-binding mAbs could block the interaction between RBD and hACE2 with an IC_50_ ranging from 0.4597 to 3.122 µg/ml (**Figure 2**).

### 3.3 Analysis of binding kinetics and binding epitope of monoclonal antibodies

**Figure 3:**
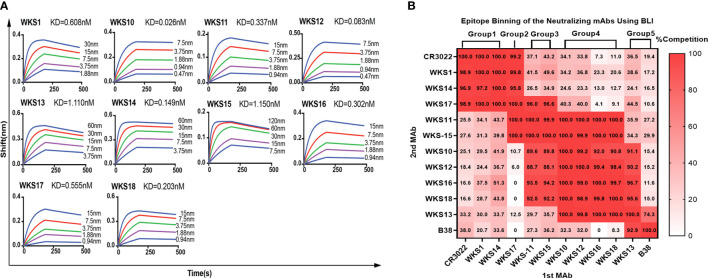
**(A)** Binding kinetics of selected anti-RBD mAbs with the recombinant ancestral RBD protein were measured by biolayer interferometry. Anti-mouse IgG Fc probes immobilized individual antibody (10 μg/mL) were saturated with serial dilutions of recombinant RBD protein (from 120 nM (18 μg/ml) to 0.47 nM (0.0705 μg/ml), as indicated). **(B)** Identification of recognizing epitopes of anti-RBD mouse mAbs by BLI with Gator™ System. The wavelength shift of the first (1st MAb) and the second (2nd MAb) antibody on the RBD protein immobilized probes were recorded and calculated as competition rates, with previously published mAbs B38 and CR3022 used as controls.

The binding kinetics of each RBD-binding mAb were measured using BLI. The binding kinetics of mAbs had low Kd values ranging from 0.026 nM (0.0039 μg/ml) to 1.150 nM (0.1725 μg/ml) (**Figure 3A**).

Next, we determined the binding epitopes of mAbs using a competitive EIA between 10 RBD-binding mAbs and 2 previously published mAbs (CR3022 and B38). mAbs that inhibit each other by >70% were considered to belong to the same group. Based on the pattern of competitive inhibition of mAb pairs, we classified our 10 RBD-binding mAbs into 5 groups (**Figure 3B**). WKS1, WKS14 and WKS17 could competitively inhibit CR3022, and therefore the epitopes of these mAbs were likely located at the inner face of the RBD (Hastie et al., 2021). WKS13 could compete with B38, and therefore likely target the receptor-binding motif (RBM) region of the RBD (Huang et al., 2022).

### 3.4 Activity of mAb against different COVID-19 variants

**Figure 4:**
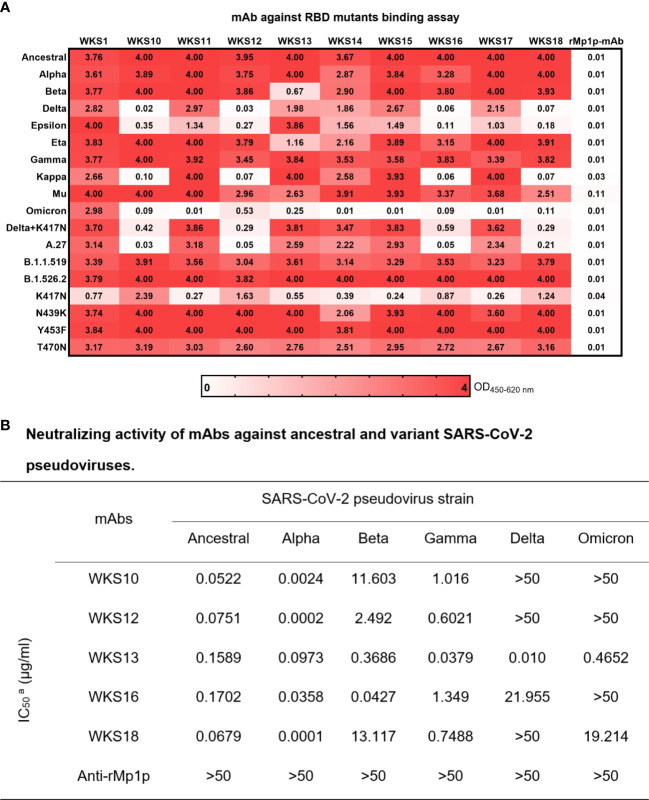
**(A)** Enzyme immunoassay for 10 anti-RBD mAbs against SARS-CoV-2 Ancestral strain and Alpha, Beta, Delta, Epsilon, Eta, Gamma, Kappa, Mu, Omicron and other RBD mutations. WKS1 and WKS13 showed broad spectrum binding to all RBDs from 0.25 to 4.00 in OD_450-620_. **(B)** Neutralizing activity of mAbs against ancestral and variant SARS-CoV-2 pseudoviruses.

The entry of SARS-CoV-2 into host cells requires the binding between SARS-CoV-2 RBD and human ACE2 receptor. Mutations in the RBD can affect the binding activity of mAbs. We first used an EIA to determine how RBD mutations affect the binding of mAb to RBD. We generated RBD that has the amino acid sequence of ancestral strain (Wuhan-Hu-1) and 13 variants including all 5 Variants of Concern (VOCs). We found that WKS1 and WKS13 were least affected by the RBD mutations and were able to bind to all 18 RBDs (**Figure 4A**).

### 3.5 Neutralizing activity against SARS-CoV-2 variants

To assess whether the mAbs can inhibit different variants, we tested the ability of WKS10, WKS12, WKS13, WKS16 and WKS18 to neutralize ancestral virus and 5 variants, including Alpha, Beta, Gamma, Delta, and Omicron, using a pVNT. Only WKS13 was able to neutralize all variants tested, with IC_50_ < 0.5 µg/ml for all variants (**Figure 4B**; **Supplementary Figure S1**).

### 3.6 Mouse WKS13 protects Syrian hamsters from SARS-CoV-2 Delta variant challenge

**Figure 5:**
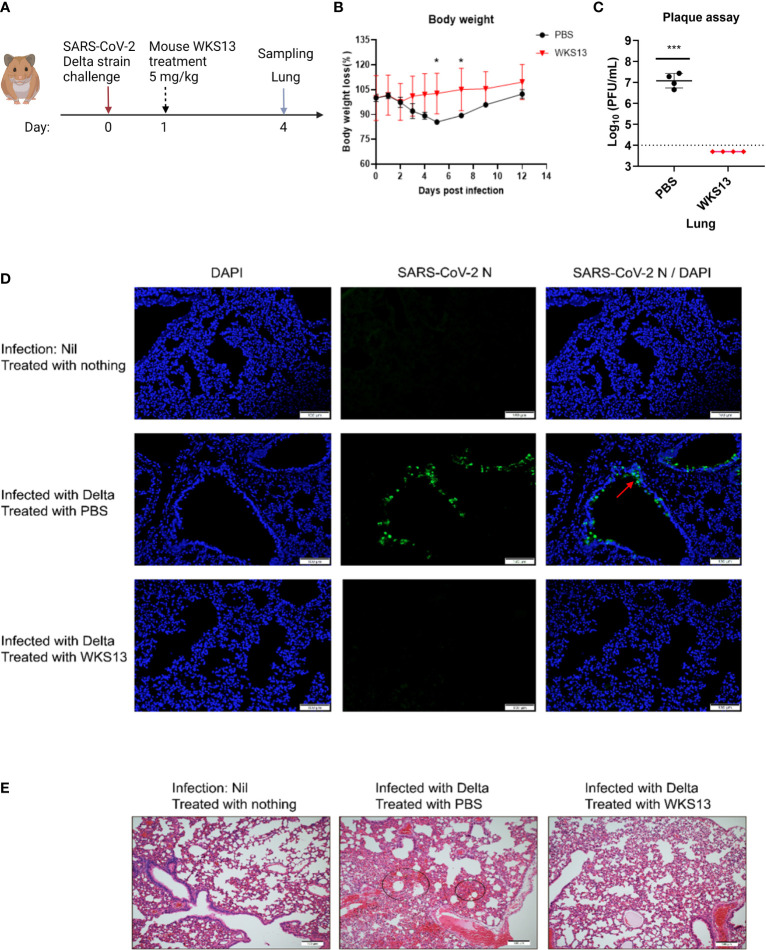
Mouse mAb WKS13 protects hamsters infected with SARS-CoV-2 Delta variant. **(A)** Schematic diagram of the experimental design. Hamsters were inoculated with 10^5^ PFU Delta variant intranasally. Twenty four hours later, these hamsters were treated with intraperitoneal injection of mouse WKS13 (5 mg/kg). On day 4 post infection, the mice were sacrificed and the lung was harvested for viral load, immunofluorescence staining and histopathological analysis. **(B)** Body weight loss. Ordinary two-way ANOVA was used for statistical analysis. **(C)** Lung viral titer as determined by plaque assay. Horizontal dotted line indicates the detection limit (10,000 PFU/ml). Ordinary two-way ANOVA was used for statistical analysis. *, P<0.05; ***, P<0.001. **(D)** Representative images of infected cells by immunofluorescence staining in lung. SARS-CoV-2 N expression (green) is shown in diffuse alveolar areas (red arrow) of PBS group, which is absent in other treatment groups. **(E)** Representative images of H&E-stained lung tissue section from hamsters treated as indicated. Scale bars, 100 μm.

Since mouse WKS13 exhibited broad-spectrum neutralizing activity *in vitro*, we tested the therapeutic effect of WKS13 in a hamster model (**Figure 5A**). Treatment with intraperitoneal administration of mouse WKS13 24 hours after Delta variant infection prevented body weight loss (**Figure 5B**) and reduced the lung viral load by about 3 log (**Figure 5C**). Immunofluorescent staining showed that the amount of virus was reduced in the lungs of WKS13-treated group when compared with the PBS group. SARS-CoV-2 N protein was abundantly expressed in the bronchioles in the control hamsters but was absent in the WKS13-treated animal lungs (**Figure 5D**). Histopathological analysis of the lung on day 4 post-infection showed multi-focal inflammation, diffuse alveolar destruction, protein-rich fluid exudate, hyaline membrane formation, marked mononuclear cell infiltration, cell debris-filled bronchiolar lumen, and alveolar collapse in the treated PBS group (**Figure 5E**). In contrast, there was a marked attenuation of the inflammatory response in the lung of WKS13-treated hamsters, with only mild pulmonary inflammatory infiltrates confined to the alveolar walls.

### 3.7 Characterization of the *in vitro* neutralizing activity and evaluation of the therapeutic effect of humanized WKS13

**Figure 6:**
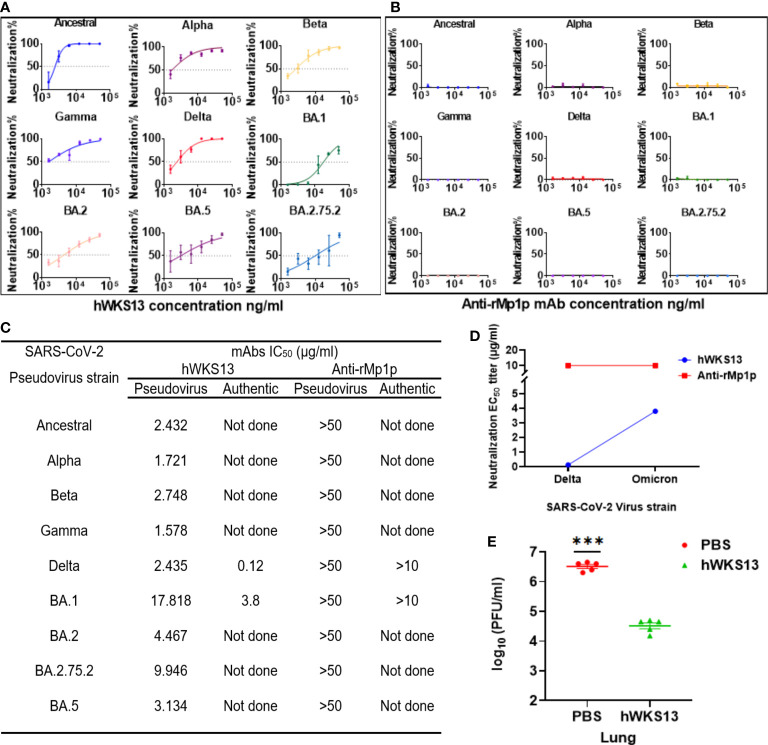
**(A, B)** Neutralizing activity of humanized WKS13 **(A)** and anti-rMp1p **(B)** against ancestral and variant SARS-CoV-2 pseudoviruses. **(C)** Neutralizing activity of humanized WKS13 and anti-rMp1p. Inhibitor vs. normalized response-variable slope was used for statistical analysis; **(D)** Evaluation of protective effect of humanized WKS13 in a hamster model. The infection and treatment scheme is same as for mouse WKS13 (**Figure 5A**). **(E)** Lung viral titer was determined using plaque assay. Ordinary two-way ANOVA and Inhibitor vs. normalized response-variable slope was used for statistical analysis. ***, P<0.001.

Since we plan to use WKS13 for use in humans, we replaced the mouse Fc fragment with human IgG1 Fc to avoid human anti-mouse antibody (HAMA) response (**Supplementary Figure S2**). The humanized WKS13 had similar neutralizing activity against ancestral virus and different variants as the original mouse WKS13 (**Figures 6A, B**). We also verified the activity of humanized WKS13 against the Delta and Omicron variants with authentic virus with cVNT (**Figures 6C, D**). In the hamster model, we showed that treatment with humanized WKS13 reduced the lung viral load (**Figure 6E**).

## 4 Discussion

mAb against the SARS-CoV-2 spike protein is one of the most effective treatment strategies for COVID-19. In this study, we derived mAbs from mice immunized with SARS-CoV-2 S protein and RBD. Out of the 960 clones screened, 18 mAbs could bind to the S protein and 5 could neutralize SARS-CoV-2. WKS13, which showed broad antiviral activities against all 5 VOCs, was selected for further evaluation. The therapeutic efficacy of WKS13, either as a murine or humanized mAb, was confirmed in a hamster model.

Seven clinically approved mAbs (Casirivimab, Imdevimab, Bamlanivimab, Etesevimab, Tixagevimab, Regdanvimab, and Bebtelovimab) have reduced efficacy against the Omicron BA.1 sublineage. Two other clinically approved mAbs (Sotrovimab and Cilgavimab) displayed a pVNT IC_50_ of 4.1 μg/ml and 2.9 μg/ml against BA.1, respectively (Bruel et al., 2022; Takashita et al., 2022), while bebtelovimab has a much lower BA.1 IC_50_ (0.007 μg/ml) (Arora et al., 2023). Our results showed that humanized WKS13 mAb was less potent against BA.1 with an IC_50_ of 17.8 μg/ml in pVNT. However, WKS13 is potent against Ancestral, Alpha, Beta, Gamma and Delta. In contrast, Casirivimab, Bamlanivimab, and Etesevimab show little efficacy against Beta and Gamma, while Bamlanivimab and Regdanvimab lost activity against the Delta variant (Guo et al., 2021; Xia et al., 2022).

Our competition assay revealed that WKS13 shares binding with B38 to the RBM. The binding ability of both WKS13 and B38 to RBM was only impacted by the K417N mutation (Hastie et al., 2021; Li et al., 2022). Therefore, the highly divergent RBM can still be the target of broad-spectrum neutralizing antibodies.

Interestingly, we noted that the binding affinity of RBD with K417N mutation only to monoclonal antibodies was weaker than that for RBD with K417N-L452R-T478K mutations (Delta + K417N) and RBD with K417N-E484K-N501Y mutations (Beta). One possibility is that L452R-T478K or E484K-N501Y mutations affected the structure of RBD, and restored the binding activity of K417N mutant to monoclonal antibodies (Ponde, 2022).

The binding affinity of WKS13 to the Omicron variant RBD is lowest among the RBDs, which is compatible with the result of the neutralizing activity. However, while there was a >16-fold difference in the binding affinity of WKS13 between Omicron and ancestral strain, the difference between the neutralizing activity was only 2.9-fold. Our data suggest that although there is a correlation between binding affinity and neutralizing activity, we cannot rely on binding affinity alone in determining the neutralizing activity.

In general, there are two main approaches in generating mAbs. First, mAb can be derived from peripheral blood mononuclear cells collected from patients recovered from COVID-19 or from vaccine recipients. Second, mAb can be generated from infected or vaccinated mice using hybridoma cells (Xia et al., 2022). The advantage of using mouse models is that these are more versatile, as recombinant proteins with different mutations can be used for generating mAbs. For example, we can use recombinant proteins that are derived from novel variants which are not yet circulating in an area, or novel variants that are circulating in animals only. Furthermore, we can use antigen pools instead of a single antigen. The rapid development and production of mAb is critical since mutations from novel variants can render a variant resistant to mAb. For example, the latest BA.5 variant is resistant to almost all currently available mAbs (Takashita et al., 2022). Furthermore, we can use potent adjuvants which may not yet be approved for clinical use. In this study, we used the novel adjuvant (QuickAntibody adjuvant), which is composed of Toll-like receptor ligands and cationic polymers (Liu et al., 2016). This adjuvant has high potency, and we could elicit a high titer of antibody even with only 3 immunizations within a span of 35 days, which is shorter than the traditional method of generating mAb in mice.

Humans develop antibody against mouse antibody. Therefore, for clinical use, it is important to use humanized version of antibody to prevent HAMA response, which can lead to reduce effectiveness of treatment or allergic reaction. In this study, humanized version of the mAb retain potent neutralizing activity *in vitro* and *in vivo*.

We have elicited the antibody response using a total of 3 doses of recombinant proteins (2 doses of spike protein and 1 dose of RBD). Our previous clinical study found that 3 doses of CoronaVac, an inactivated vaccine containing the ancestral strain, could elicit neutralizing antibodies against the Omicron BA.2 variant (Fong et al., 2023). Whether a heterologous priming with the entire spike protein and boosting with only the RBD induce broader neutralizing antibody response than homologous 3-dose RBD remains to be determined.

There are several limitations in this study. First, we only evaluated WKS13 *in vivo* using the Delta variant challenge. This is because Omicron variant is much less virulent in the hamster model (Shuai et al., 2022; Suzuki et al., 2022), and hence the protective effect of WKS13 cannot be readily evaluated for Omicron variant. Second, although we have demonstrated that WKS13 likely shared similar binding epitopes as B38 in the competitive EIA assay, further structural analysis is required to determine the exact location of the binding epitope. Third, although we have replaced the murine monoclonal antibody Fc fragment with the human IgG1 Fc fragment, its safety in human requires further clinical studies. Fourth, since SARS-CoV-2 is continuously evolving, the novel variants may have additional mutations that escape from WKS13 neutralization.

## 5 Conclusion

In summary, we have successfully derived potent and broad-spectrum therapeutic mAbs against SARS-CoV-2 using a versatile spike/RBD-vaccinated mouse hybridoma approach. These mAb, either alone or in combination of other mAbs, represent potential treatment options for COVID-19 patients.

## Data availability statement

The original contributions presented in the study are included in the article/**Supplementary Material**, further inquiries can be directed to the corresponding author/s.

## Ethics statement

The study was conducted in accordance with the Declaration of Helsinki, and approved by Animal Ethics Committee on the Use of Live Animals in Teaching and Research of HKU (CULATR) (protocol code 5730-20 and approval date 31-March-2020).

## Author contributions

Conceptualization, KWe, J-PC, HZ, K-YY and KK-WT. Methodology, KWe, J-PC, XF, XZ, CL, K-MT, HS, L-LC, RZ, JS, H-WT, and KWa. Writing-original draft preparation, KWe, J-PC and KK-WT. Writing-review and editing: SY, K-YY, JF-WC. Supervision: HZ, KK-WT. Funding acquisition, K-YY, JF-WC, HZ, KK-WT. All authors have read and agreed to the published version of the manuscript.
