# Supplementary figures and images for: Broad-spectrum humanized monoclonal neutralizing antibody against SARS-CoV-2 variants, including the Omicron variant
Source: Front Cell Infect Microbiol. 2023 Aug 14;13:1213806. doi: 10.3389/fcimb.2023.1213806 (PMC10461085; doi:10.3389/fcimb.2023.1213806)

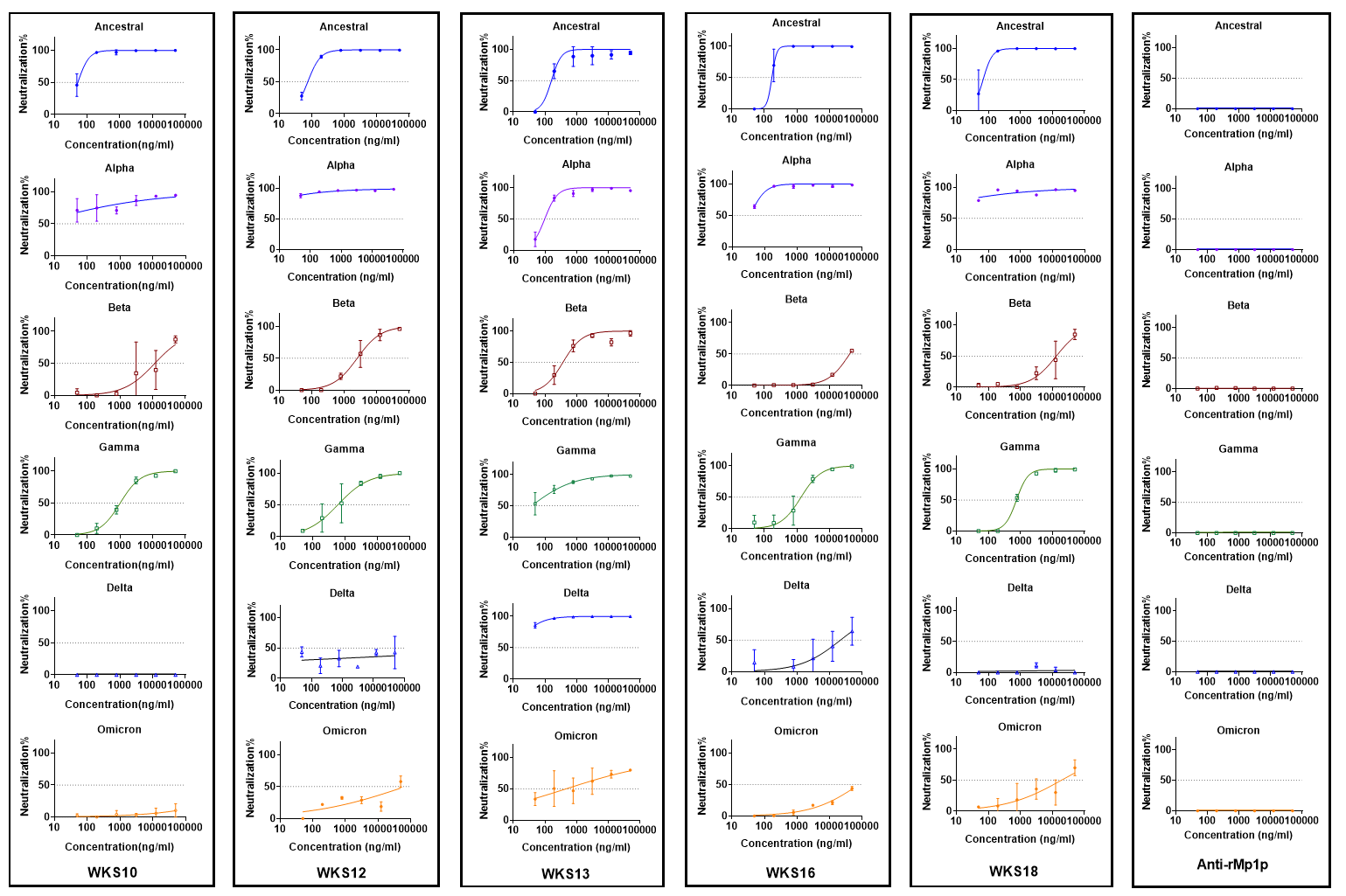

Supplement: Supplementary file 1 [file Image_1.tif]

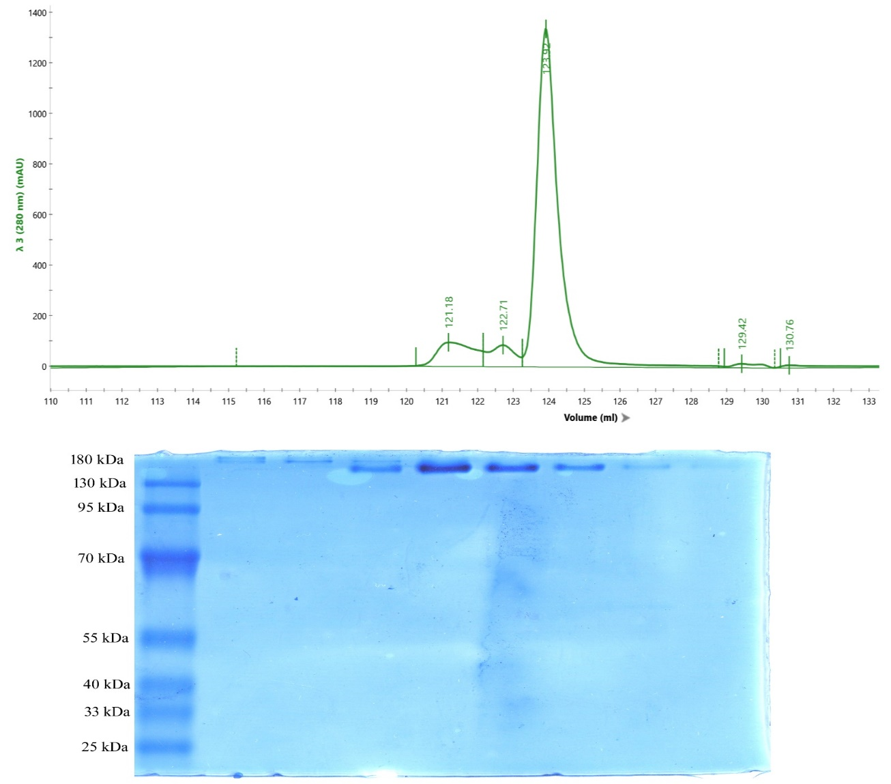

Supplement: Supplementary file 2 [file Image_2.tif]
