# Supplementary material for: Broad-spectrum humanized monoclonal neutralizing antibody against SARS-CoV-2 variants, including the Omicron variant
Source: Front Cell Infect Microbiol. 2023 Aug 14;13:1213806. doi: 10.3389/fcimb.2023.1213806 (PMC10461085; doi:10.3389/fcimb.2023.1213806)
Supplement: Supplementary file 3 [file Table_1.docx]

**Supplementary Table S1.** Amino acid mutations in the receptor binding domain of SARS-CoV-2.

| **RBD Variants** | **Amino acid mutations^a^ of the spike gene** |
| --- | --- |
| Ancestral | No mutations |
| Alpha | N501Y |
| Beta | K417N, E484K, N501Y |
| Delta | L452R, T478K |
| Epsilon | L452R |
| Eta | E484K |
| Gamma | K417T, E484K, N501Y |
| Kappa | L452R, E484Q |
| Mu | R346K, E484K, N501Y |
| Omicron | G339D, S371I, S373P, S375F, S477N, T478K, E484A, Q493R, G496S, Q498R, N501Y, N505H |
| Delta+K417N | K417N, L452R, T478K |
| A.27 | L452R, N501Y |
| [B.1.1.519](https://cov-lineages.org/lineage.html?lineage=B.1.1.519) | T478K |
| [B.1.526.2](https://cov-lineages.org/lineage.html?lineage=B.1.526.2) | S477N |
| Single mutation | K417N |
| Single mutation | N439K |
| Single mutation | Y453F |
| Single mutation | T470N |

^a^ Compared with Wuhan-Hu-1.
