# Supplementary material for: Broad-spectrum humanized monoclonal neutralizing antibody against SARS-CoV-2 variants, including the Omicron variant
Source: Front Cell Infect Microbiol. 2023 Aug 14;13:1213806. doi: 10.3389/fcimb.2023.1213806 (PMC10461085; doi:10.3389/fcimb.2023.1213806)
Supplement: Supplementary file 4 [file Table_2.docx]

**Supplementary Table S2**. Amino acid mutations in the spike of SARS-CoV-2 pseudoviruses

| **Pseudovirus variant** | **Amino acid mutations^a^ of the spike gene** |
| --- | --- |
| Ancestral | No mutations |
| Alpha | Delete 69-70, delete 144-145, N501Y, A570D, D614G, P681H,T716I, S982A, S1118H |
| Beta | D80A, D215G, delete 241-243, K417N, E484K, N501Y, D614G, A701V, |
| Gamma | L18F, T20N, P26S, D138Y, R190S, K417T, E484K, N501Y, D614G,H655Y, T1027I, V1176F |
| Delta | T19R, E156G, delete 157-158, L452R, T478K, D614G, P681R, D950N |
| BA.1 | A67V, delete 69-70, T95I, G142D, delete 143-145, N211I, delete 212, 214 insect EPE, G339D, S371I, S373P, S375F, S477N, T478K, E484A, Q493R, G496S, Q498R, N501Y, N505H, T547K, D614G, H655Y, N679K, P681H, N764K, D796Y, N856K, N969K, L981F |
| BA.2 | T19I, L24S, delete 25/27, G142D, V213G, G339D, S371F, S373P, S375F, T376A, D405N, R408S, S413R, N440K, S477N, T478K, E484A, Q493R, Q498R, N501Y, Y505H, D614G, H655Y, N679K, P681H, K714N, N764K, D796Y, Q954H, N969K |
| BA.2.75.2 | T19I, L24S, delete25/27, G142D, K147E, W152R, F157L, I210V, V213G, G257S, G339H, R346T, S371F, S373P, S375F, T376A, D405N, R408S, K417N, N440K, G446S, N460K, S477N, T478K, E484A, F486S, Q498R, N501Y, Y505H, D614G, H655Y, N679K, P681H, N764K, D796Y, Q954H, N969K, D1199N |
| BA.5 | T19I, L24S, delete 25/27, delete 69/70, G142D, V213G, G339D, S371F, S373P, S375F, T376A, D405N, R408S, K417N, N440K, L452R, S477N, T478K, E484A, F486V, Q498R, N501Y, Y505H, D614G, N679K, P681H, N764K, D796Y,Q954H, N969K |

^a^ Compared with Wuhan-Hu-1
