# Supplementary material for: Broad-spectrum humanized monoclonal neutralizing antibody against SARS-CoV-2 variants, including the Omicron variant
Source: Front Cell Infect Microbiol. 2023 Aug 14;13:1213806. doi: 10.3389/fcimb.2023.1213806 (PMC10461085; doi:10.3389/fcimb.2023.1213806)
Supplement: Supplementary file 5 [file Table_3.docx]

**Supplementary Table S3**. Spike/RBD binding and neutralizing activity of the 18 selected monoclonal antibodies in this study.

| **mAb** | **Subtype** | **EIA OD_450-620_^a^** | | **Able to neutralize SARS-CoV-2**^b^ |
| --- | --- | --- | --- | --- |
|  |  | Against S protein | Against  RBD |  |
| WKS1 | IgG2b | **1.4390** | **2.6110** | - |
| WKS2 | IgG1 | **3.4425** | 0.0108 | - |
| WKS3 | IgG1 | **3.2835** | 0.0072 | - |
| WKS4 | IgG2a | **3.7250** | 0.0164 | - |
| WKS5 | IgG2a | **3.5865** | 0.0098 | - |
| WKS6 | IgG1 | **3.4190** | 0.0189 | - |
| WKS7 | IgG1 | **3.4500** | 0.0099 | - |
| WKS8 | IgG3 | **0.7860** | 0.0130 | - |
| WKS9 | IgG2b | **2.7535** | 0.0116 | - |
| WKS10 | IgG2b | **3.4040** | **3.0500** | + |
| WKS11 | IgG2a | **2.8056** | **2.3760** | - |
| WKS12 | IgG1 | **3.0105** | **2.5665** | + |
| WKS13 | IgG1 | **2.8795** | **2.4450** | + |
| WKS14 | IgG1 | **2.2605** | **1.6180** | - |
| WKS15 | IgG2a | **2.4710** | **1.6400** | - |
| WKS16 | IgG1 | **3.2290** | **2.5210** | + |
| WKS17 | IgG1 | **1.3345** | **1.3870** | - |
| WKS18 | IgG1 | **3.3135** | **2.4165** | + |

**Abbreviations**: EIA, enzyme immunoassay; RBD, receptor binding domain; S protein, spike protein; +, can neutralize SARS-CoV-2 (ancestral strain); -, cannot neutralize SARS-CoV-2 (ancestral strain)

^a^ The OD_450-620_ was measured with mAbs at a concentration of 0.5 μg/ml. The cutoff OD_450-620_ values for positivity for S protein and RBD are 0.0609 and 0.0462, respectively. Monoclonal antibodies with binding activity to S protein or RBD are highlighted in bold.

^b^ mAb concentration at 3.125 μg/ml
